# *Oestrus ovis* Nasal Myiasis with Pupation in Human Host, Greece, October 2025

**DOI:** 10.3201/eid3203.251077

**Published:** 2026-03

**Authors:** Ilias P. Kioulos, Emmanouil Kokkas, Evangelia-Theophano Piperaki

**Affiliations:** Agricultural University of Athens, Athens, Greece (I.P. Kioulos); University of Crete, Heraklion, Greece (E. Kokkas); Foundation for Research and Technology Hellas, Institute of Molecular Biology and Biotechnology, Heraklion (E. Kokkas); National and Kapodistrian University of Athens Medical School, Athens (E.T. Piperaki)

**Keywords:** Oestrus ovis, sheep bot fly, parasites, nasal, myiasis, third instar, L3, puparium, pupation, Greece

## Abstract

We report a case of human *Oestrus ovis* nasal myiasis in Greece, in which pupation occurred within the human host. Ten larvae in various stages of development and 1 puparium were expelled or extracted from the patient’s maxillary sinus. Diagnosis was confirmed through morphologic identification and by PCR, followed by DNA sequencing.

*Oestrus ovis* (Diptera: Oestridae), the sheep bot fly, is a cosmopolitan parasite of small ruminants, widespread in hot and dry regions, including countries bordering the Mediterranean Sea. Accidental human infestations by *O. ovis* flies have been reported from around the world (*1*,*2*). We report a case of human *O. ovis* nasal myiasis in which pupation occurred within the human host. The diagnosis was confirmed by molecular identification of the parasite using PCR followed by DNA sequencing.

The patient was a 58-year-old woman in Greece who worked outdoors on a Greek island, adjacent to a field with grazing sheep. It was September, during hot and dry weather, and she noticed numerous flies swarming around her face. Approximately 1 week later, she had onset of progressive maxillary pain, followed over the next 2–3 weeks by severe coughing. She reported no other symptoms. On October 15, she sought medical attention after she sneezed and “worms” started coming out of her nose. An otolaryngologist surgically removed 10 larvae of various stages and 1 pupa from her maxillary sinus. She was treated with nasal decongestants and made a complete recovery. None of her co-workers reported similar symptoms.

We examined 2 of the larvae and part of a puparium (Figure). The puparium fragment was ≈10 mm long, black, and wrinkled and contained remnants of the pupa. One larva was yellowish, measured ≈15 mm, and exhibited rows of spines ventrally, with a bare preanal bulge; its posterior peritremes were circular with a central button and no distinct suture. The second larva measured ≈20 mm, was light brown, and displayed broad transverse blackish bands dorsally. On the basis of size and morphology, we identified both larvae as third instar (L3) *O. ovis* bot fly.

**Figure:**
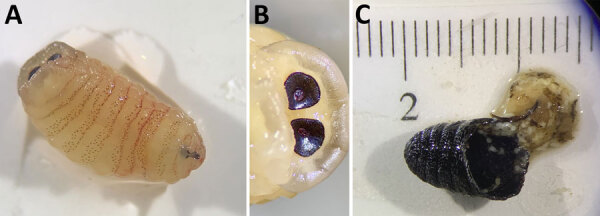
Third instar *Oestrus ovis* larva and puparium retrieved from nasal sinuses of a 58-year-old female patient, Greece. A) The third instar was yellowish, with rows of spines on the ventral surface. B) The posterior peritremes were circular with a central button. C) The broken puparium was black and wrinkled and contained remnants of the pupa.

We extracted genomic DNA from the pupal casing by using DNAzol (Molecular Research Center, https://www.mrcgene.com) according to the manufacturer’s instructions. We amplified a 392-bp fragment of the mitochondrial cytochrome c oxidase I gene and a 190-bp fragment of the ribosomal DNA 28S gene, as previously described (*3*). Both PCR reactions produced specific bands at the expected sizes. We purified the products by using the GeneJet PCR Purification Kit (Thermo Fisher Scientific, https://www.thermofisher.com) and sequenced them (GENEWIZ, https://www.genewiz.com). Sequencing results demonstrated 100% identity of the amplified fragments with their corresponding GenBank sequences (accession no. KX268655.1 for *cox1* and KP974974.1 for 28S rDNA). We performed alignments by using the ClustaW function in BioEdit version 7.2.5 (https://thalljiscience.github.io).

Myiasis can be classified ecologically as obligatory (caused by larvae that require a living vertebrate host), facultative (caused by free-living larvae that may opportunistically develop in hosts), and accidental (caused by free-living larvae, unable to complete their life cycle in a host). Anatomically, myiasis is categorized by affected site as sanguinivorous, cutaneous (furuncular or migratory), wound, or cavitary (e.g., cerebral, aural, nasal, or ophthalmomyiasis) (*1*).

The *O. ovis* life cycle within its natural hosts, sheep and goats, is well-documented. The female deposits first instar (L1) larvae into the animal’s nostrils, which migrate upward into the nasal passages and paranasal sinuses, where they feed, grow, and molt. They are expelled as third instar (L3) larvae, burrow into the soil, and pupate. *O. ovis* bot flies infrequently affect humans, most often depositing larvae in the conjunctival sac and rarely into the nostrils, mouth, or external auditory meatus. The most common clinical manifestation is acute catarrhal conjunctivitis, typically preceded by the sudden sensation of a foreign body (*1*). Until recently, it was believed that *O. ovis* larvae could not develop beyond the L1 stage in humans. In recent years, however, L2 (*4*) and L3 (*5*,*6*) larvae have been recovered from human case-patients, typically in the setting of immunosuppression or in patients with traumatic or anatomic abnormalities of the nasal passages. Of the 5 reported cases of *O. ovis* myiasis in travelers returning from Greece, 4 involved L1 (*7*–*10*) and 1 involved L2 larvae (*4*).

The patient we report had a severely deviated nasal septum and appears to have been inoculated with a large larval burden. From a purely anatomic perspective, we hypothesize that the combination of high larval numbers and septum deviation impeded normal egress from the nasal passages, permitting progression to the L3 stage and, in 1 instance, pupation. Of note, L3 larvae that become trapped within the sinuses of animals are not known to pupate; instead, they desiccate, liquefy, or calcify, with occasional bacterial superinfection. Pupation of *O. ovis* larvae within any mammalian host is considered biologically implausible. The paranasal sinus environment does not meet temperature and humidity requirements for pupation, and host secretions, immune responses, and resident microbiota create a hostile milieu for pupal development. In our patient, unidentified anatomic or physiologic factors within the paranasal sinuses, probably including her severe septum deviation, apparently permitted pupation. Alternatively, this case may represent an early indication of evolutionary adaptation, enabling *O. ovis* parasites to complete their life cycle in humans. In either scenario, additional cases and data are needed to understand this phenomenon, but clinicians should be aware of the potential for human bot fly infections in endemic areas.
